# Reprogramming to pluripotency does not require transition through a primitive streak-like state

**DOI:** 10.1038/s41598-017-15187-x

**Published:** 2017-11-29

**Authors:** Stefanie Raab, Moritz Klingenstein, Anna Möller, Anett Illing, Jelena Tosic, Markus Breunig, Georg Kuales, Leonhard Linta, Thomas Seufferlein, Sebastian J. Arnold, Alexander Kleger, Stefan Liebau

**Affiliations:** 1 Institute of Neuroanatomy & Developmental Biology (INDB), Eberhard Karls University Tuebingen, Oesterbergstr. 3, 72074 Tuebingen, Germany; 2 Department of Internal Medicine I, University Medical Center Ulm, Albert-Einstein-Allee 23, 89081 Ulm, Germany; 3 Institute of Experimental and Clinical Pharmacology and Toxicology II, Faculty of Medicine, University of Freiburg, Freiburg, Germany; 4 Spemann Graduate School of Biology and Medicine (SGBM), Albert-Ludwigs-University Freiburg, Freiburg, Germany; 5 Faculty of Biology, Schänzlestraße 1, Albert-Ludwigs-University Freiburg, Freiburg, Germany; 6 BIOSS Centre of Biological Signalling Studies, Albert-Ludwigs-University, Freiburg, Germany

## Abstract

Pluripotency can be induced *in vitro* from adult somatic mammalian cells by enforced expression of defined transcription factors regulating and initiating the pluripotency network. Despite the substantial advances over the last decade to improve the efficiency of direct reprogramming, exact mechanisms underlying the conversion into the pluripotent stem cell state are still vaguely understood. Several studies suggested that induced pluripotency follows reversed embryonic development. For somatic cells of mesodermal and endodermal origin that would require the transition through a Primitive streak-like state, which would necessarily require an Eomesodermin (*Eomes*) expressing intermediate. We analyzed reprogramming in human and mouse cells of mesodermal as well as ectodermal origin by thorough marker gene analyses in combination with genetic reporters, conditional loss of function and stable fate-labeling for the broad primitive streak marker *Eomes*. We unambiguously demonstrate that induced pluripotency is not dependent on a transient primitive streak-like stage and thus does not represent reversal of mesendodermal development *in vivo*.

## Introduction

During mammalian development, early cell fate decisions during the process of gastrulation lead to the formation of the three germ layers, namely ectoderm, mesoderm and endoderm. The development of mesoderm and definitive endoderm (DE) is initiated by cell rearrangements of pluripotent, epithelial cells of the posterior epiblast and a subsequent epithelial-to-mesenchymal transition (EMT) leading to the formation of the primitive streak (PS). Mesoderm and DE cells are recruited as they migrate through the PS, while epiblast cells which do not ingress through the streak give rise to neuroectodermal progeny including the epidermis and central nervous system (reviewed in^1^). PS formation is initiated and maintained by a complex network of transcription factors and signaling pathways. Signals include feed forward loops of *WNT*, *TGFβ* and *BMP* factors involving reciprocal tissue interactions of epiblast and trophectoderm (reviewed in^1^). Absence or misexpression of these signals in the epiblast leads to an impaired PS formation followed by disorganization or absence of the mesoderm and endoderm germ layers and embryonic lethality. Examples are null mutants for Nodal or *Wnt* signaling, such as *Wnt3* ^2^, the nuclear receptor *Nr5a2* ^3^, or the T-box transcription factor Eomesodermin (Eomes)^4,5^.

The specification of different PS-derived cell types follows a strict spatio-temporal pattern. The most anterior PS gives rise to the early transient population of mesendoderm cells that contribute to the DE and axial mesoderm. This population is followed by cells ingressing through the anterior third of the streak generating the anterior mesoderm that gives rise to head and cardiogenic mesenchyme and extraembryonic mesoderm^4,5^. Cells ingressing at more posterior streak levels are giving rise to paraxial, intermediate and lateral plate mesoderm. The signaling pathways regulating streak patterning include Nodal- and Wnt-activities. For example, high levels of Nodal activities induce the expression of mesendodermal marker genes such as *Eomes, Mixl1, Tdgf1* (Cripto), *Lhx1 and Foxh1* (reviewed in^6,7^). In particular, *Eomes* is critically required for the specification of early mesendoderm (DE and anterior mesoderm)^1,4,8^, and all cells of the early PS transiently express Eomes. *Eomes*-deficiency in the epiblast also dramatically perturbs PS formation due to defective EMT leading to early embryonic arrest^4,8^. Similarly, the sequential formation of different cellular subtypes of the streak can be mimicked by i*n vitro* cell differentiation and monitored by the expression of marker genes. The *in vitro* differentiation of pluripotent stem cells towards the three germ layers can be guided by similar signaling stimuli and mRNA expression profiles usually reflect the *in vivo* situation^9,10^.

During reprogramming to induced pluripotency through forced expression of the core pluripotency factors *SOX2, OCT4, KLF4, and C- MYC*, somatic cells lose their differentiated state^11,12^. Several reports suggested that reprogramming follows distinct stages resembling a reversal of embryonic development^13,14^. Fibroblasts, as the most common starting cell type for reprogramming, represent cells of mesoderm origin. Thus, the reversal of their cellular ontogeny during reprogramming would most likely involve the passage through a PS-like stage^14^. Accordingly, it was proposed that a sequential cascade of EMT–MET facilitates the reprogramming process^13,15^.

To initiate the transcription factor networks and signaling pathways that are characteristic for pluripotent cells, extensive alterations in the epigenetic landscape take place such as broad changes of chromatin modifications, chromatin architecture, and gross changes in the cellular transcriptome^16^. Although several studies have explored mechanisms and stages during the reprogramming process^17^, the question concerning an analogy of the reprogramming process as reversal of physiological embryonic development, including gastrulation is controversial^14^. Moreover, it is debatable why also cells derived from ectodermal lineages, such as astrocytes or keratinocytes would show a PS-like global gene expression pattern during reprogramming^14^, given that ectodermal cells developmentally never ingress through the PS. Thus, it is questionable, whether these events indeed reflect reverted embryonic development or might rather represent changes in transcriptional programs induced by the forced expression of reprogramming factors. To address these developmental aspects of reprogramming, we used different reprogramming approaches including somatic cells from different germ layers and organisms, namely murine and human cells as well as different reporter alleles and fate analysis tools. Thereby, we provide evidence that somatic cell reprogramming neither follows a reversed mesendoderm development nor that occurring mesendodermal gene signatures reach physiological and functionally relevant levels during differentiation.

## Results

### Gene expression patterns during reprogramming of human somatic cells of mesoderm and ectoderm origin

**Figure 1:**
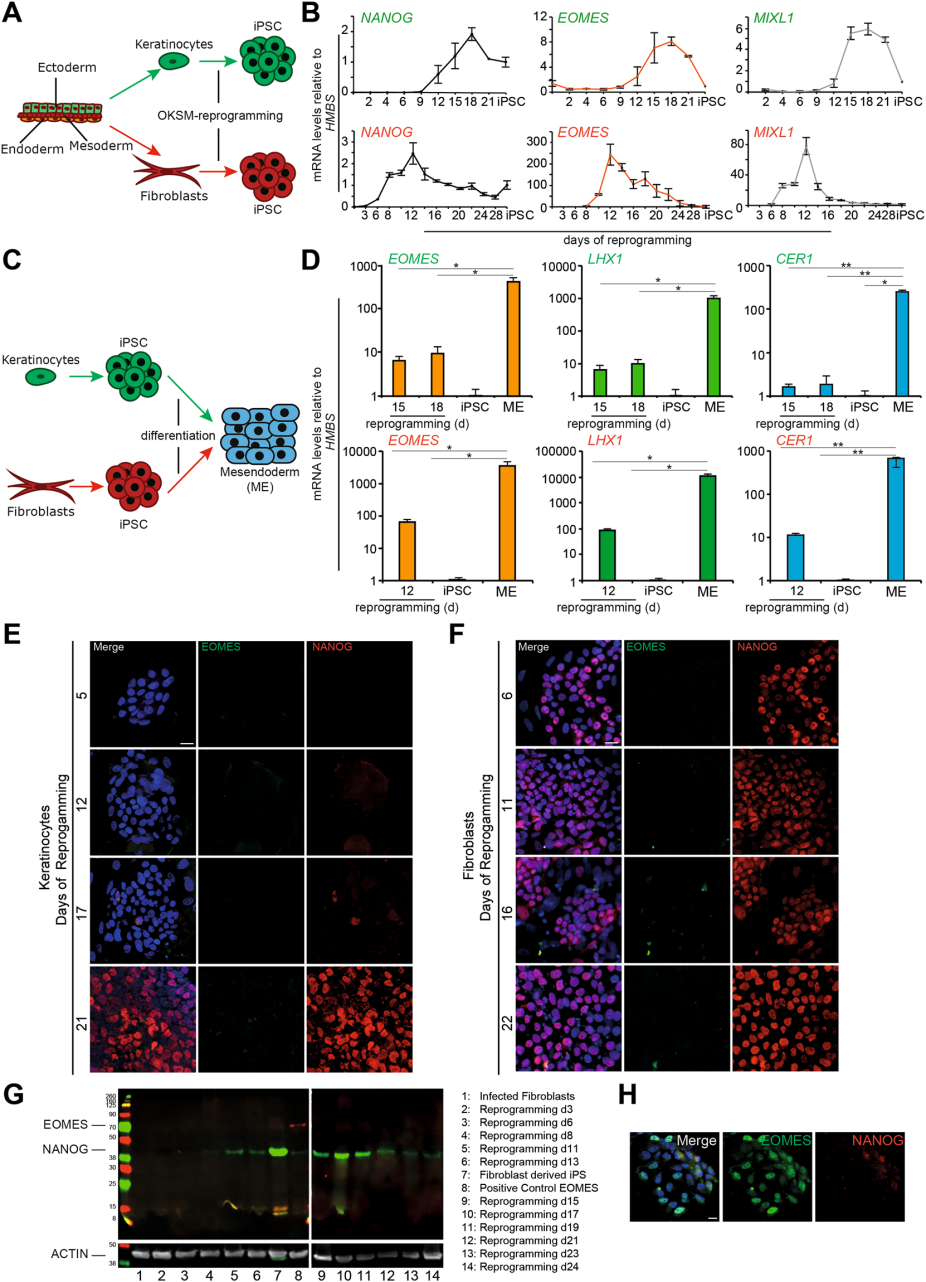
Upregulation of primitive streak and mesendoderm markers during reprogramming of human somatic cells of fibroblast and keratinocyte origin, but absence of EOMES protein. (**A**) Schematic overview of the reprogramming experiment for the somatic cells of ectodermal –keratinocytes, and mesodermal origin – fibroblasts. (**B**) Expression patterns of indicated genes during reprogramming of human keratinocytes (upper row) and human fibroblasts (lower row). All mRNA levels are expressed relative to the housekeeping gene HMBS and values have been normalized to iPSCs, which have been set to 1 to illustrate fold induction. (**C**) Schematic overview of the experimental setup of mesendoderm differentiation. (**D**) Comparison of marker gene expression for definitive endoderm (*CER1*) and mesendoderm/primitive streak (*LHX1, EOMES*) are lower in magnitude for the reprogrammed cells compared to the differentiated cells. (**E,F**) Protein expression of EOMES and NANOG during the time course of human keratinocytes (**E**) and human fibroblasts (**F**) reprogramming. The scale bar represents 100 µm for all images. (**G**) Western Blot analysis EOMES and NANOG protein expression during reprogramming of fibroblasts. Actin was used as loading control. Lane 1: human foreskin fibroblasts (HFFs) protein lysate, lane 2–6 and 9–15: consecutive days of HFFs reprogramming at indicated time points (d = day), lane 7: fibroblast derived iPSCs, lane 8: mesendodermal differentiation as a positive EOMES control. **(H)** Mesendodermal differentiation of iPSCs shows a positive signal for EOMES protein.

To investigate if cells during human reprogramming follow stages of reversed embryonic development, we transduced keratinocytes and fibroblasts which have ectodermal and mesodermal origin, respectively, with a polycistronic *OKSM (OCT3/4, KLF4, SOX2, c-MYC*) construct to monitor and directly compare gene expression signatures during reprogramming (Fig. 1A, Supplemental Fig. 1A). Consistent with previous reports^14^, a transcriptional signature resembling a PS-like and mesendodermal program was observed during reprogramming of both cell types representing different germ layer origin (Fig. 1B, Supplemental Fig. 1B,C). Expression patterns of key markers of PS formation and subsequent early mesendoderm differentiation (*EOMES*, *T*, *CER, LHX1, FGF4, FGF8, MIXL1*) were similarly regulated in both keratinocytes and fibroblasts. However, reprogramming of keratinocytes appeared delayed compared to fibroblasts as shown by the expression profile of the pluripotency marker *NANOG* (Fig. 1B). Of note, particularly *NANOG* expression, previously shown to reinforce mesendoderm differentiation during pluripotency exit^18^, coincides with the mesendodermal signature (Fig. 1B, Supplemental Fig. 1B,C). Both mesodermal fibroblasts and ectodermal keratinocytes displayed an increase in mesendoderm and primitive streak markers starting at day 6–8 (fibroblasts) or 9–12 (keratinocytes) with a peak between day 12–14 (fibroblasts; except for *CER1*) or 15–18 (keratinocytes), followed by the downregulation of these genes (except *FGF8*) until the induced pluripotent stem cell (iPSC) state (Fig. 1B, Supplemental Fig. 1B,C). Next, we aimed to determine the expression range of this mesendodermal gene signature by comparing mRNA levels of cells during reprogramming with cells undergoing directed mesendoderm differentiation *in vitro*. (Fig. 1C). This direct comparison showed that mRNA levels of mesendoderm markers (*EOMES, LHX1, CER1*) were several magnitudes higher in differentiating cells compared to the expression during reprogramming (Fig. 1D). Given that *EOMES* expression is critical for PS and subsequent mesendoderm formation^1,4,8^, we evaluated EOMES protein levels during reprogramming. However, no EOMES protein was detected, neither by immunocytochemistry nor by Western blot (in fibroblasts), during reprogramming of fibroblasts and keratinocytes (Fig. 1E–G). As a control, expression of the pluripotency marker NANOG was analyzed in parallel showing increasing protein levels during reprogramming (Fig. 1E–G). As control, mesendodermal differentiation of hiPSCs displayed the expression of EOMES protein on day 3 as analyzed by immunofluorescent staining (Fig. 1H). In summary, these results indicate that PS and mesendoderm markers are significantly upregulated during the course of reprogramming. At the same time NANOG reaches its expression peak, suggesting that the establishment of pluripotency networks triggers a PS-like expression phenotype. However, mRNA levels are detected at much lower levels compared to those found during mesendodermal differentiation (Fig. 1D).

### Murine fibroblasts undergoing reprogramming do not express Eomes protein

**Figure 2:**
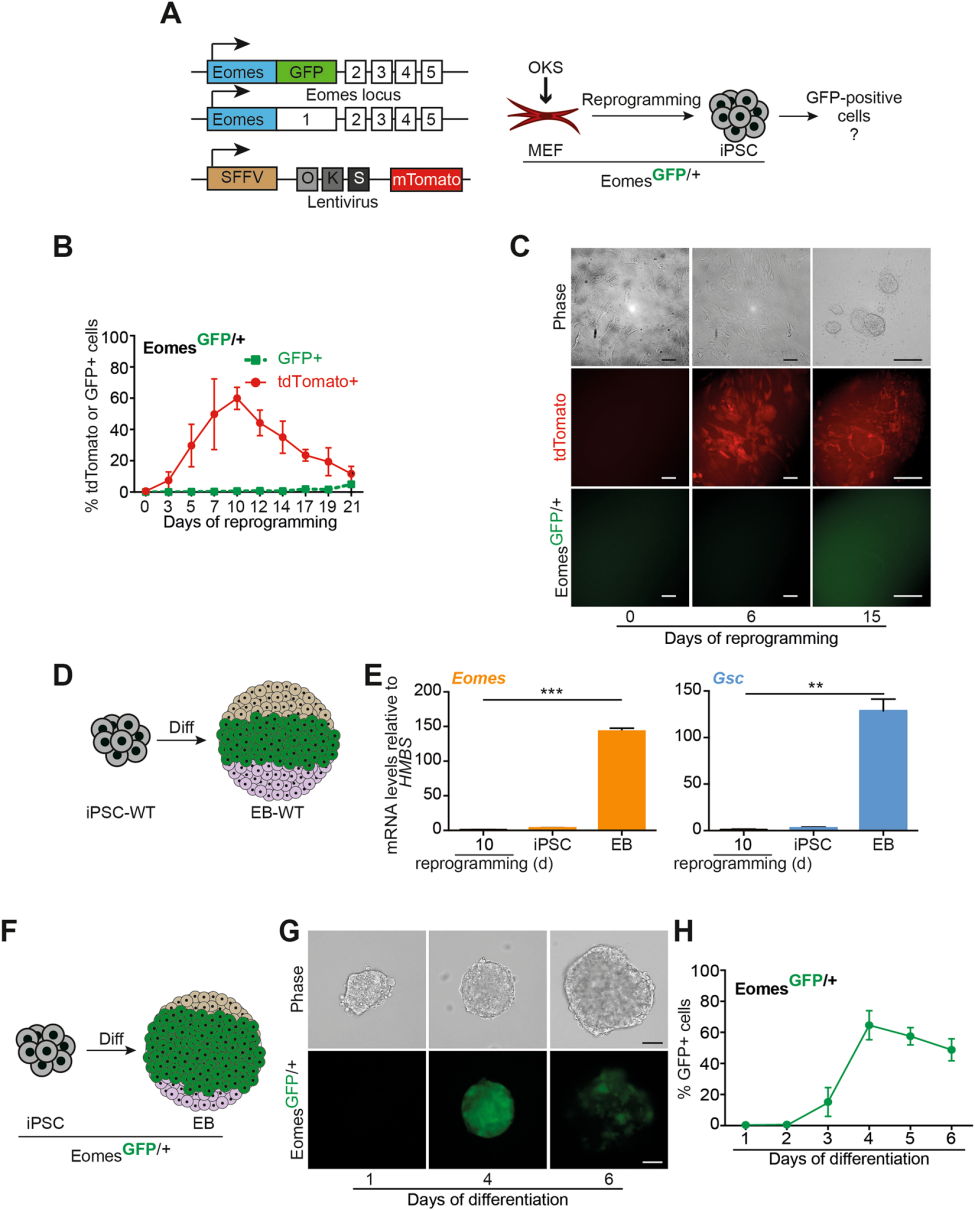
EOMES protein is not detectable during several stages of murine fibroblast reprogramming. (**A**) Schematic illustration of the *Eomes* alleles used in (**B–H**). MEFs carry a GFP knock-in at the *Eomes* ^GFP/+^ locus (upper and middle panel) allowing for quantification of *Eomes* ^GFP/+^ positive cells by putative GFP expression^19^. A lentiviral 3-factor (OKS) reprogramming construct includes a tdTomato reporter to track expression of pluripotency markers during reprograming^20^. (**B**) FACS-based quantification of GFP- and tdTomato-positive cells during reprogramming at the indicated days. GFP-positive cells arise only late, after the reprogramming process indicating differentiation of formed iPSCs (day 21). (**C**) Corresponding phase contrast images (upper panel) and fluorescence images of the MEF cultures during reprogramming (red, middel panel) and the *Eomes* ^GFP/+^ reporter signal (green, lower panel). (**D**) Scheme for spontaneous *in vitro* differentiation of WT-iPSCs towards embryoid bodies representing early germ layer formation mirrored in the three colors. (**E**) Comparison of marker gene expression for mesendoderm expression peaks during reprogramming of murine fibroblasts in comparison to iPSCs and cells differentiated in EBs. All mRNA levels are expressed relative to the housekeeping gene Hmbs and values have been normalized to day 10 reprogramming cultures, which have been set to 1 to illustrate fold induction. (**F**) Scheme for *in vitro* differentiation of *Eomes* ^GFP/+^ reporter iPSCs isolated from (**A**) in embryoid bodies toward mesendoderm using high doses of Activin A. Germ layer formation is mirrored in the three colors, while high doses of Activin A favor endoderm formation (green). (**G**) *Eomes* ^GFP/+^ reporter iPSCs are differentiated towards mesendoderm. Expression of GFP validates the functionality of the *Eomes* ^GFP/+^ reporter. (**H**) FACS-based quantification of independent experiments from (**G**).

To corroborate the finding that PS markers are frequently expressed only at low mRNA level during reprogramming, independent of the parental germ layer origin, we investigated the expression of *Eomes* as one of the key TFs for PS and mesendoderm development^1,4,8^. Since we didn’t detect EOMES protein during human somatic cell reprogramming (Fig. 1E–G), we sought to apply mouse embryonic fibroblasts (MEFs) harboring a *Eomes* ^GFP/+^ reporter allele^19^ to track eventually arising *Eomes*-expressing, GFP-positive cells using very sensitive FACS techniques (Fig. 2A). *Eomes* ^GFP/+^ MEFs were transduced with a polycistronic OKS (*OCT3/4, KLF4, SOX2*) construct harboring a Td-tomato expression cassette to visualize cells that undergo reprogramming^20^ (Fig. 2A,B). During 21 days of reprogramming, FACS analyses and immunocytochemistry for both the GFP and Tomato signal were conducted at intervals of 2 or 3 days. FACS-analysis did not reveal any GFP-positive cells, while the red tomato-signal from the reprogramming cassette expectedly got silenced when reaching the iPSC state on day 21^20^ (Fig. 2B,C). In line with human data, we observed *Eomes* and other mesendodermal marker up-regulation on mRNA level but in a far lower range than observed in spontaneous, differentiating mouse iPSC cultures (Supplemental Fig. 2; Fig. 2D,E). To control for the efficiency of the *Eomes* ^GFP/+^ reporter, we differentiated resulting *Eomes* ^GFP/+^ iPSCs using high doses (50 ng/ml) of Activin A to drive mesendoderm formation and could detect high levels of GFP-expression. Thus, we could successfully validate the functionality of the reporter allele during differentiation (Fig. 2F–H). Despite the inability to detect *Eomes* ^GFP/+^ reporter expression during reprogramming, transient Eomes expression cannot be entirely excluded, as cell samples were harvested at time-intervals of 2–3 days.

### Eomes expression remains undetectable during cell lineage tracing

**Figure 3:**
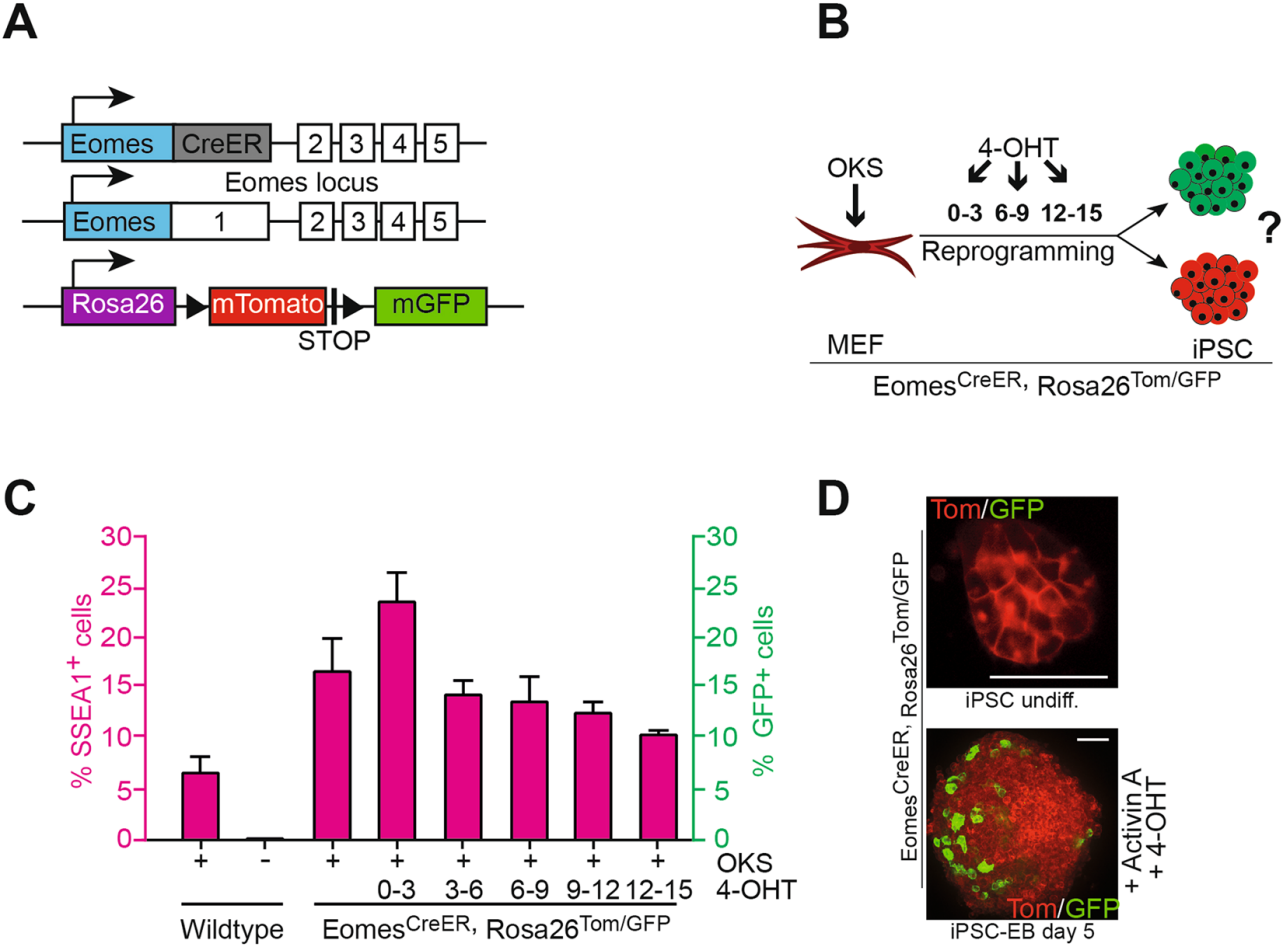
Eomes protein remains absent upon lineage tracing during MEF reprogramming. (**A**) Schematic illustration of the alleles used in (**C,D**). In one *Eomes* allele, the promoter drives a tamoxifen-inducible Cre-recombinase, while the ROSA26-locus harbors a floxed Tom/GFP color switch-reporter. *Eomes* ^CreER/+^-recombinase activity deletes the tomato leading to GFP activation and a subsequent color switch from red to green^21^. (**B**) Schematic showing lineage tracing approach to test whether iPSCs pass through an *Eomes*-positive state. (**C**) FACS-analysis for SSEA1- and GFP-positive cells at day 18 of reprogramming. Tamoxifen (4-OHT) treatment as indicated. Note that neither condition generates GFP-expressing cells. Representative experiment from n = 2 in triplicates is shown. (**D**) Upper image: representative image of *Eomes* ^CreER/+^ROSA26^Tom/GFP^-iPSCs (isolated from experiments in (**C**) after 4-OHT treatment) further substantiates data obtained in (**C**). Lower image: day 5 embryoid body generated from E*omes* ^CreER/+^ROSA26^Tom/GFP^-iPSCs upon 4-OHT and Activin A treatment contains GFP-positive cells. Note that the arising iPSCs from (**C**) and upper image (**D**) remain GFP-negative independent of 4-OHT treatment, while 4-OHT and Activin A treatment of established lines induces the expected color switch, indicating specificity of the lineage-tracing allele *in vitro*. Scale bars in all images: 50 µm.

To rigorously test if *Eomes* is significantly expressed at any stage during reprogramming, we used MEFs carrying a 4-hydroxytamoxifen (4-OHT)-inducible CreERT in the *Eomes* locus (Eomes^CreERT^) and a Cre-inducible fluorescent reporter cassette (Rosa26^Tom/GFP^) to permanently lineage-label *Eomes*-expressing cells^21^ (Fig. 3A,B). Ssea1 was used in FACS analysis to mark pluripotent cells and asses the efficiency of reprogramming. Among the detected Ssea1-positive cells, no GFP-positive cells were detected after tamoxifen treatment on days 3–15 during reprogramming (Fig. 3C). The absence of GFP-positive cells in successfully reprogrammed cells was further confirmed by immunofluorescence in picked and expanded iPSC cultures (Fig. 3D, upper image), indicating the lack of *Eomes* expression during and at the end of the reprogramming process. To validate the linage labeling tool used in these experiments, we induced mesendoderm differentiation of resulting iPSCs in the presence of tamoxifen and Activin A, which resulted in the appearance of GFP-positive, Eomes expressing cells within the differentiating Tomato-positive embryonic bodies (Fig. 3D, lower image).

### Eomes is dispensable for reprogramming of somatic cells of mesoderm origin

**Figure 4:**
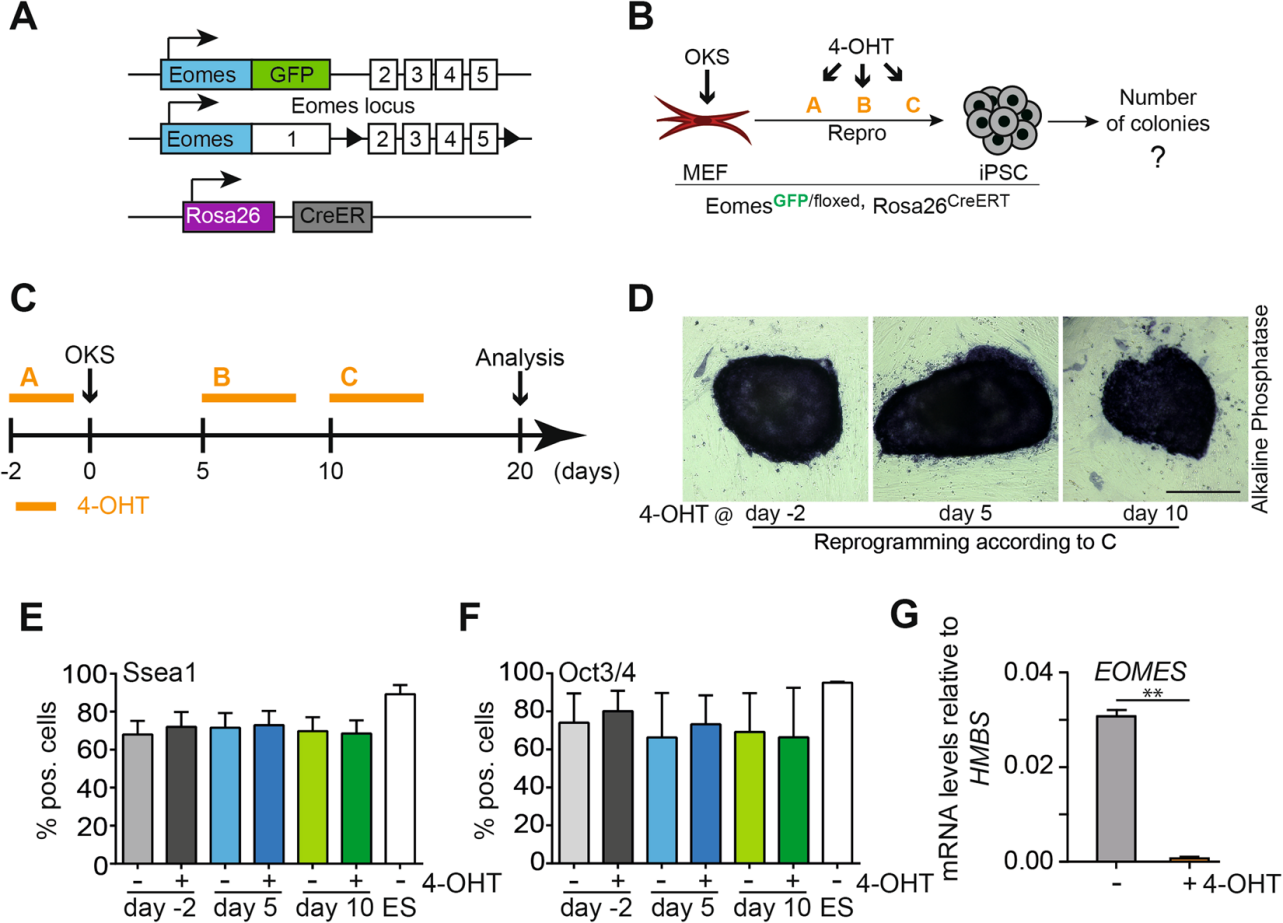
Eomes is dispensable for reprogramming of murine fibroblasts. (**A**) Schematic illustration of the *Eomes* alleles used in (**D**–**G**). MEFs carry one functional null allele with a GFP knock-in at the *Eomes* locus and a second conditional allele, where exons 2–5 are flanked by loxP sites. The tamoxifen (4-OHT)-inducible CreER-recombinase is expressed from the Rosa26 locus and used to induce the complete genetic deletion of *Eomes* by 4-OHT administration^8^. (**B**,**C**) 4-OHT treatment regimen used for timed *Eomes* ablation during reprogramming. Orange lines indicate tamoxifen treatment intervalls: A: d-3 to d-1 (48 h), B: d5–9 (96 h), C: d10-d14 (96 h). (**D**) Representative images of Alkaline phosphatase staining of iPSC colonies at different timepoints of 4-OHT treatment as indicated. (**E**,**F**) FACS-based quantification of (**E**) Ssea1 and (**F)** Oct3/4 positive cells at day 20 of reprogramming following with and without 4-OHT administration. Scale bars in all images: 50 µm. Representative experiments from n = 3 in triplicates are shown.

To exclude that very low amounts of Eomes protein are being expressed at levels undetectable via FACS or immunofluorescent staining, we used MEFs carrying the *Eomes* ^GFP^ reporter allele and a floxed *Eomes* allele^8^ (*Eomes* ^GFP/fl^) in combination with a ubiquitously expressed 4-hydroxytamoxifen (4-OHT)-inducible CreERT (Rosa26^CreERT^) to inducibly delete *Eomes* function during reprogramming (Fig. 4A,B). Given the critical role of Eomes for PS formation in the early embryo, we reasoned that the reprogramming of cells lacking *Eomes* expression would be impaired if the transition of cells through the PS-like intermediate state would be a crucial step during the reprogramming process. Cells were transduced with the OKS construct^20^ and treated with tamoxifen at different time points to induce the genetic deletion of *Eomes* (orange letters; Fig. 4B,C). Efficient reprogramming was assessed by alkaline phosphatase staining (Fig. 4D), FACS staining for Ssea1-positive cells (Fig. 4E) and Oct3/4 expression (Fig. 4F). The loss of *Eomes* did not result in any significant change in number, morphology, or marker expression of arising iPSC colonies, irrespective of the time-point of induced deletion (Fig. 4D-F), albeit the slight *Eomes* expression peak (Supplemental Fig. 2) could be ablated upon tamoxifen treatment (Fig. 4G). This indicates that *Eomes* is functionally entirely dispensable for the reprogramming to pluripotency, despite its prominent role during PS formation and gastrulation initiation.

## Discussion

Cellular events during reprogramming were extensively studied over the past years. However, the different stages during reprogramming need to be further defined and exact molecular mechanisms remain to be resolved. Several studies of changes in gene expression during reprogramming have suggested that cells undergo a reversal of embryonic development including MET-EMT events and transiently acquire a PS-like gene expression signature^13,14,15^. Gene expression patterns of PS formation, as well as EMT-MET events, occur independently of an epithelial or mesenchymal origin of the starting cell population during reprogramming^13,14,15^. Thus, it remains questionable whether reprogramming indeed follows stages of “reverse embryonic development”, or if observed gene signatures solely represent the spurious activation of developmental programs, or if genetic programs are indeed necessary to establish the pluripotency network. The latter view was recently supported by studies indicating that EMT-related transcription factors cooperate with core factors of the pluripotency circuitry to induce pluripotency^22,23^. Here, we apply different genetic tools including fate-analysis and reporter alleles at the *Eomes* gene locus, as one of the central transcription factors with important functions in the gastrulating mouse embryos for PS formation, EMT and specification of the mesendoderm lineages. None of the applied genetic tools and analyses indicated significant expression of Eomes on the route to iPSC reprogramming. Additionally, we tested if *Eomes* was functionally required during reprogramming by genetically deleting *Eomes* in starting cells. Indeed, the genetic deletion of *Eomes* had no effect on reprogramming efficiency, suggesting that the induction of a transient PS-like state is no crucial step during reprogramming.

We propose that observed mesendodermal/PS-like expression profiles in cells that are undergoing reprogramming reflect a non-physiological transcriptional response to the reprogramming factors. Thus, it is likely that high level expression of reprogramming factors by the lentiviral transduction and subsequent re-activation of the pluripotency network triggers the transcription of mesendodermal genes. In addition to maintaining the pluripotent state, transcription factor of the pluripotency network such as *Nanog, Oct3/4, Klf4, and Tbx3* share functions during the early phase of exit from pluripotency. Thus, they contribute to the initiation of transcriptional programs to guide mesendoderm cell fate determination, e.g. by regulating *Eomes* expression^5,24–26^. In that way, core pluripotency factors govern the first steps of differentiation and cell fate determination^5,26,27^. This hypothesis is underlined by the fact that mesendoderm transcriptional signatures can be likewise found in cells of ectodermal origin during reprogramming, although these cells never go through a mesendodermal/PS-like state during *in vivo* embryogenesis. Finally, the extent of mesendodermal gene transcription levels did not reach the range of physiological lineage differentiation as shown for murine and human somatic cell reprogramming. In summary, our data confirm the previously described mesendodermal fingerprint arising during reprogramming in cells of mesoderm and ectoderm origin^14^. While previous reports interpreted these findings as a reversed process of embryonic development^13–15^, our data instead favors a non-physiological transcriptional response resulting from the forced induction of the pluripotency network, which does not reflect the magnitude of gene expression as seen during mesendodermal lineage commitment *in vivo* ^5,24–26^.

## Material and Methods

### Cell cultures

Rat embryonic fibroblasts (REFs) from embryonic day 14 Sprague Dawley rats were generated according to the protocol previously described in^28^ and were cultured in Dulbecco’s Modified Eagle’s Medium (DMEM, Sigma Aldrich) containing 10% fetal bovine serum (FBS, Sigma Aldrich/Biochrom), 1% GlutaMAX, 1% nonessential amino acids (NEAA), and 1% antibiotic-antimycotic (all from Life Technologies). REFs were treated with 7.5 μg/mL mitomycin C (Biomol) for 2.5 hours for mitotic inactivation. All animal experiments were performed in compliance with the guidelines for the welfare of experimental animals issued by the Federal Government of Germany, the National Institutes of Health and the Max Planck Society. The experiments in this study with respect to generation of MEFs were approved by the review board of the Land Baden-Wuerttemberg, Permit Number Nr. O.103. HEK293T cells for lentivirus production were cultured in DMEM supplemented with 1% penicillin/streptomycin (P/S, Sigma Aldrich) and 10% FBS.

### Cells for reprogramming

Mouse embryonic fibroblasts (MEFs) were cultured according to standard methods at 5% CO2 and 37 °C as described previously in^29,30^. Briefly, DMEM was supplemented with 15% FBS, 1% P/S, 1% GlutaMAX, 1% NEAA, 1 mM Sodium Pyruvate (Sigma Aldrich), 1% β‐Mercaptoethanol (Merck Millipore) and 0.05 mg/ml Vitamin C. The cultivation of keratinocytes from plucked human hair was performed according to^28,31–33^. In brief, keratinocytes were cultured on 20 μg/mL collagen IV (Sigma Aldrich) coated dishes in EpiLife medium with HKGS supplement (Gibco® Life Technologies) until they reached about 70% confluency. Human foreskin fibroblasts (HFFs) (System Biosciences) were cultivated in DMEM supplemented with 10% FBS and 1% GlutaMAX, 1% NEAA, and 1% antibiotic-antimycotic.

### Cultivation of induced pluripotent stem cells (iPSCs)

Human iPSCs were cultured on Matrigel-coated (Corning) 6-well plates in FTDA culture medium at 5% CO2, 5% O2 as described in^34–37^. Mouse iPSCs were cultured either in feeder-dependent conditions or in feeder-free conditions (2i)^38^. For feeder-dependent conditions (ES feeder medium) Knockout^TM^ DMEM (KO-DMEM; Life-Technologies) was supplemented with 15% FBS, 1% P/S, 1% GlutaMAX, 1% NEAA, 1% Sodium Pyruvate, 1% β-Mercaptoethanol and 240 U/ml leukaemia inhibitory factor (LIF, Cell guidance systems). In case of feeder-free (2i) culture mouse KO-DMEM with 15% Knockout Serum Replacement (KOSR, Life Technologies), 1% P/S, 1% GlutaMAX, 1% NEAA, 1% Sodium Pyruvate, 1% β-Mercaptoethanol, 240U/ml LIF and 1 μM PD0325901 (Calbiochem) and 3 μM GSK3β-inhibitor CHIR99021 (Axon Medchem) was used.

### Lentivirus production

Lentivirus production and vector systems encoding for human and mouse variants were described previously^20,28,29^.

### Generation of induced pluripotent stem cells (iPSCs)

#### Human

Human iPSCs were generated from plucked human hair keratinocytes and from human foreskin fibroblasts (System Biosciences). Keratinocytes were cultured and infected as described in^32^. Fibroblasts were cultured in DMEM, 10% FBS, 1% antibiotic-antimycotic, 1% NEAA, and 1% GlutaMAX. For reprogramming 1*10^5^ fibroblasts were plated on coated 6-well plates and were infected with 5*10^8^ viral copies of STEM CCA^39^ OKSM lentivirus on two subsequent days in culture medium supplemented with 10 µM Rock inhibitor/Y-27632 (Selleckchem), 8 µg/ml polybrene (Sigma Aldrich). On the third day infected keratinocytes and fibroblasts were distributed equally into 6-well plates on mitomycin-inactivated rat embryonic fibroblast (REF) feeder cells. 1,5*10^4^ REFs were mitotically inactivated with 7,5 µg/ml mitomycin C for 2,5 h. During reprogramming cells were cultured in KO-DMEM, 20% KOSR, 1% antibiotic-antimycotic, 100 μM NEAA, 1% GlutaMAX, 50 mM β-mercaptoethanol, 50 μg/ml L-Ascorbic acid (Carl Roth), 10ng/ml FGF2 (Cell Guidance Systems), 10 µM Rock inhibitor/Y-27632 (Selleckchem) at 5% CO2, 5% O2, and 37 °C, and medium was changed every second day. IPSC colonies were mechanically transferred onto Matrigel coated (Corning) 6-well plates after three weeks.

#### Mouse

MEFs were seeded on a gelatine coated plate (4 × 10^4^ cells/12-well) one day prior to infection. Next day, 5 µl concentrated polycistronic OKS (OCT3/4, KLF4, SOX2) lentivirus harboring a Td-tomato^20^ together with 8 µg/ml polybrene (Sigma Aldrich) in 1 ml ES Feeder Medium was added to each 12-well. After 8 h of incubation at 37 °C, medium was removed, cells were washed with PBS and ES-Feeder medium was added and refreshed daily. At day 6, medium was changed to ES Feeder KOSR, where FCS was exchanged by KOSR. On day 20, cells were either stained for alkaline phosphatase (AP) expression according to standard protocols or cells were analyzed by flow cytometry.

### Differentiation of human iPSCs

For mesendodermal differentiation, iPSCs (70% confluency) were incubated with basal medium RPMI Media 1640, 1% antibiotic-antimycotic, 1% GlutaMAX, 2% B-27 Supplement (all Thermo Fisher) supplemented for the first day with 500 nM IDE1 (StemCell Technologies), 50ng/ml BMP4 (PeproTech), 3 µM CHIR-99021 (Selleckchem), 5 µM LY294002 (Selleckchem) at 5% CO2, 5% O2 and 37 °C. For the next three days 500 nM IDE1, 50ng/ml BMP4, 5 µM LY294002, 20ng/ml FGF2 (Cell Guidance Systems) was added to the basal medium.

### Differentiation of mouse iPSCs

Cells were seeded in hanging drops (400cells/20 µl) in N2B27 medium. N2B27 medium was produced by adding 37,5 ml IMDM, 12,5 ml Ham’s F12 medium, 0.5x B27, 0.5x N2 (all Gibco® Life Technologies), 1% P/S, 0.05% BSA, 1% GlutMAX, 2 mM Ascorbic acid, and 450 µM 1-thioglycerol (Sigma). After two days, drops were washed off with 5 ml N2B27 Medium containing 50ng/ml Activin A (PreproTech) and transferred to non-adherent plates. Medium was changed every 48 h by carefully centrifuging the cells at 800 rpm for 2 min, discarding the supernatant up to about 500 µl and carefully resuspending the embryoid bodies in fresh medium (N2B27 + Activin A) on a new plate.

### Tamoxifen treatment

4-Hydroxytamoxifen (Sigma Aldrich) was added to the cell culture medium to a final concentration of 1 µg/ml for the respective time frames. After tamoxifen treatment cells were washed with PBS once and received fresh medium.

### Immunocytochemistry

Cells were fixed using 4% paraformaldehyde, 10% sucrose for 15 min on ice. All subsequent steps were performed at room temperature. After washing twice 5 min with PBS (Thermo Fisher) cells were permeabilized with 0.2% TritonX (Carl Roth) for 5 min. Following blocking for 1.5 h with 5% normal donkey serum (NDS, Sigma Aldrich), samples were incubated with primary antibodies for 2 h. The primary antibody Sheep α-Eomes (R&D Systems AF6166) was diluted 1:28, Rabbit α-Nanog (Cell Signaling 9656) 1:200. After washing with PBS, samples were incubated with 1:1000 diluted Alexa Fluor®-labeled secondary antibodies α-Sheep 488 nm (Abcam ab15077) and α-Rabbit 568 nm (Abcam ab175470) for 1 h. Following a final washing step with PBS, samples were mounted using ProLong® Gold Antifade with DAPI (Thermo Fisher). All images were captured using Axio Imager M2 microscope and analyzed using AxioVision software (Zeiss).

### Western Blot

Western Blotting was performed according to standard protocols. In brief, cell pellets were lysed in cold RIPA-Puffer (150 mM NaCl, 50 mM Tris-HCl (pH 8.0), 5 mM EDTA (pH 8.0), 1% IGEPAL® (Sigma Aldrich), 0.5% Na-Deoxycholat, 0.1% SDS, 1X Halt™ Protease and Phosphatase Inhibitor Cocktail (Thermo Fisher)). The samples were loaded on a 12% SDS Mini-PROTEAN TGX Precast Protein Gel (Bio-Rad) and subsequently blotted on a nitrocellulose membrane (Protran). Before adding the primary antibodies (Sheep α-Eomes (1:28) (R&D Systems AF6166), Rabbit α-Nanog (1:200) (Cell Signaling 9656), Chicken αnti ß-Actin (1:1000) (Abcam ab13822), samples were blocked using 6% porcine serum in 1x TBS buffer for 2 h. Primary antibodies were diluted in blocking solution and incubated overnight at 4 °C. For subsequent washing steps 0.05% Tween20 in 1x TBS was used. Secondary antibodies (α-Sheep, α-Rabbit, α-Chicken 800 CW or 680RD (LI-COR)) were diluted as recommended from the manufacturer and incubated for 1.5 h in the dark. Membrane was developed using a near-infrared fluorescence system, Odyssey® FC and analyzed using Image Studio Lite software (LI-COR).

### Gene expression Analysis

Total RNA was isolated from cell lysates using RNeasy Mini Kit according to the manufacturer’s instructions (Qiagen). First, cDNA synthesis was performed using 80 ng RNA with RT Buffer (Promega), dNTPs (GE Healthcare), Hexanucleotide Mix (Roche) and MMLV RT (Promega). For the preamplification step PreAmp Master Mix, SuperScript III First-Strand Synthesis SuperMix (both Thermo Fisher), TE buffer (Ambion) was used according to the manual. To quantify the amount of the genes of interest QuantiTect Primer Assays (Qiagen) were used on the BioMark HD System with 96.96 Dynamic Arrays (both Fluidigm). Relative gene expression was calculated as a ratio of target gene concentration to the housekeeping gene concentration. Details have been described in^38,40^.

### FACS analysis

While Ssea1 surface staining and Tomato/GFP auto-fluorescence staining was performed on living cells, cells were fixed in 4% paraformaldehyde, 10% sucrose for 20 minutes on ice for intranuclear Oct3/4 staining. Stainings were performed according to standard methods. Briefly, adherent cells were washed with PBS and dissociated into single cell suspension by incubation with 0.25% trypsin/EDTA (Millipore). For staining of cells were blocked in PBS supplemented with 10% FBS, incubated with primary antibody α-Ssea1 (1:1600) (Cell Signaling MC480) for 1.5 h on ice in the dark, and incubated with secondary antibody α-mouse AlexaFluor 647 nm (1:600) (Invitrogen A21238) for 30 min on ice in the dark. Washing steps were performed with PBS with 2% FBS, and 1% P/S (FACS Buffer). For staining of Oct3/4 paraformaldehyde-fixation was followed by permeabilization of cells for 30 min in 0.5% Saponin (Sigma Aldrich) in FACS Buffer, blocking in 5% normal goat serum (Sigma Aldrich), and 0.5% Saponin in PBS for 20 min on ice. Antibodies were diluted in the blocking dilution, the primary antibody α-Oct3/4 (Santa Cruz sc-5279) 1:100, and the secondary antibody α-mouse AlexaFluor 488 nm (Invitrogen A11029) 1:200. Washing steps were performed with FACS Buffer supplemented with 0.5% Saponin. Cells were analyzed with a FACSAria II or III flow cytometer (BD). All events were gated with forward scatter and side scatter profiles.

### Statistical analysis

All experiments were independently repeated at least 3 times and Error bars in the graphs show calculated Standard Error if not otherwise stated. Statistical significance was calculated using Students t-test. p-values have been calculated where appropriate and now illustrated by asterisks according to the following definitions: *p < 0.05; **p < 0.01; ***p < 0.001. GraphPad Prism 5 was used for statistical and graphical data evaluations.

## Electronic supplementary material

Supplementary Material and Figures
