# Supplementary material for: Reprogramming to pluripotency does not require transition through a primitive streak-like state
Source: Sci Rep. 2017 Nov 29;7:16543. doi: 10.1038/s41598-017-15187-x (PMC5707390; doi:10.1038/s41598-017-15187-x)

Reprogramming to pluripotency does not require transition through a primitive streak-like state

Stefanie Raab[1#], Moritz Klingenstein[1#], Anna Möller[2#], Anett Illing[2], Jelena Tosic[3,4,5], Markus Breunig[2], Georg Kuales[3], Leonhard Linta[1], Thomas Seufferlein[2], Sebastian J. Arnold[3,6$], Alexander Kleger[2$], Stefan Liebau[1$]

[1] Institute of Neuroanatomy & Developmental Biology (INDB), Eberhard Karls University Tuebingen, Oesterbergstr. 3, 72074 Tuebingen, Germany

[2] Department of Internal Medicine I, University Medical Center Ulm, Albert-Einstein-Allee 23, 89081 Ulm, Germany

[3] Institute of Experimental and Clinical Pharmacology and Toxicology II, Faculty of Medicine, University of Freiburg, Freiburg, Germany

[4] Spemann Graduate School of Biology and Medicine (SGBM), Albert-Ludwigs-University Freiburg, Freiburg, Germany

[5] Faculty of Biology, Schänzlestraße 1, Albert-Ludwigs-University Freiburg, Freiburg, Germany

[6] BIOSS Centre of Biological Signalling Studies, Albert-Ludwigs-University, Freiburg, Germany.

[#] contributed equally

[$] contributed equally

Corresponding authors:

Dr. Sebastian J Arnold
Email: sebastian.arnold@pharmakol.uni-freiburg.de
Phone: +49-761-203-96819
Fax: +49-731-203-96820
Institute of Clinical and Experimental Pharmacology and Toxicology
Albertstr. 25
79104 Freiburg, Germany

Prof. Dr. Alexander Kleger
Email: alexander.kleger@uni-ulm.de
Phone: +49-731-500-44728
Fax: +49-731-500-44665
Department of Internal Medicine I, University Medical Center Ulm
Albert-Einstein-Allee 23
89081 Ulm, Germany

Prof. Dr. Stefan Liebau
Email: stefan.liebau@uni-tuebingen.de
Phone: +49-7071-2978233
Fax: +49-7071-294014
Institute of Neuroanatomy & Developmental Biology (INDB), Eberhard Karls University Tübingen
Österbergstr. 3
72074 Tübingen

Supplemental figure legend

Supplemental figure 1: Expression profiles of human somatic cells during reprogramming display primitive streak and mesendoderm signatures

(A) Schematic overview of the epithelial-to-mesenchymal transition (EMT) and the early steps of human embryonic development. The blastocyst includes the first differentiated cells in human development – the pluripotent inner cell mass or embryoblast and the trophoblast. The pluripotent embryoblast cells differentiate to the so called germinal disc, composed of epiblast and hypoblast. Gastrulation starts when cells of the epiblast form the primitive streak undergo EMT and migrate between hypo- and epiblast. The cells integrating and exchanging the former hypoblast give rise to the endoderm. The cells between the two disks differentiate to mesoderm, while the remaining cells in the epiblast have ectodermal fate. Keratinocytes are known to differentiate from the ectoderm while fibroblasts are of mesoderm origin. (**B**,**C**) Expression patterns during reprogramming of fibroblasts (**B**) keratinocytes (**C**). Single marker gene profiles for genes expressed during primitive streak and mesendoderm formation in reprogramming keratinocytes and fibroblasts towards pluripotency are shown.

Supplemental figure 2: Expression profile for Eomesodermin (Eomes) during reprogramming of mouse embryonic fibroblasts. Time points as indicated.

Supplemental Figure 1

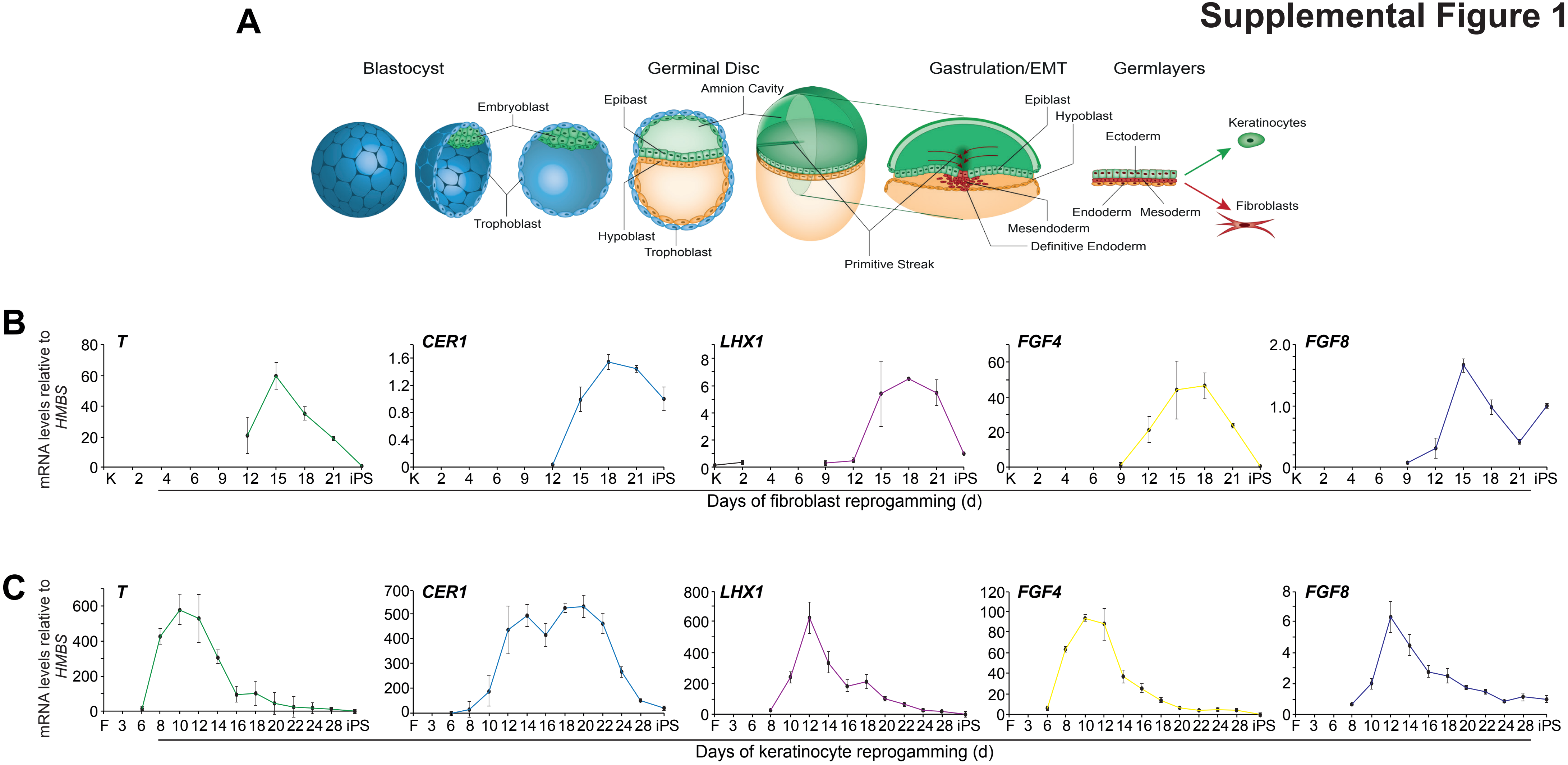

Supplemental Figure 2

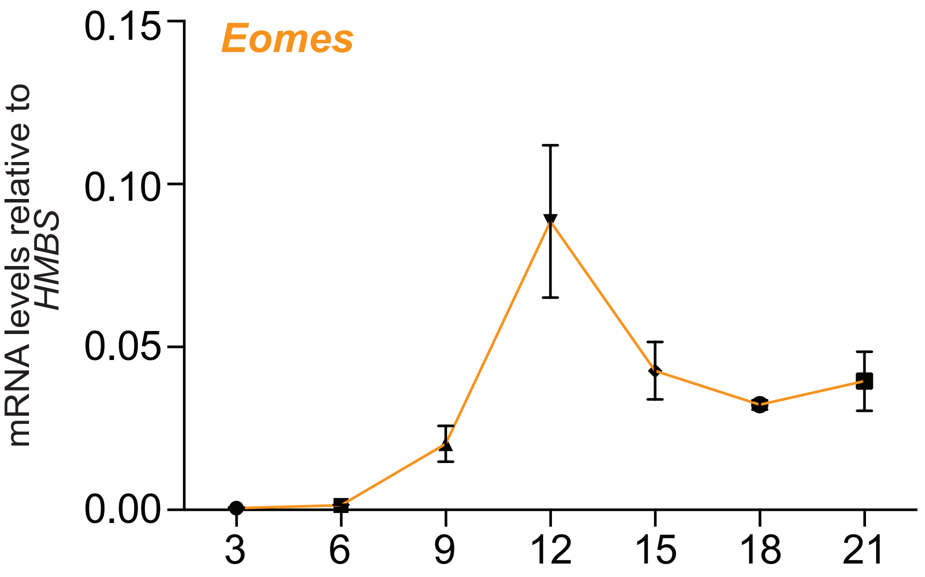

Supplement: Supplementary file 1 — Supplementary Material and Figures [file 41598_2017_15187_MOESM1_ESM.pdf]
